# Reviews of Books

**Published:** 1922-09

**Authors:** 


### REVIEWS OF BOOKS.

**INTRODUCTORY LECTURES ON PSYCHO-ANALYSIS.** By Prof. Sigmund Freud, M.D., LL.D. Translated by Joan Riviere. (George Allen & Unwin, Ltd.; Pp. 395; 18s. net)

The mass of literature devoted to the exposition of the psycho-analytic doctrine may be evidence of its value—or it may be an indication that it is so vague and hypothetical that it is easy to write a book to prove anything by it. Where knowledge is precise we do not find this. Thus, until it was proved that the essential factor in the causation of general paralysis of the insane is the spirochæte of syphilis there was an extensive literature of all sorts of theories. Until careful research reveals more and more definitely the nature of normal and of abnormal mental processes we shall continue to be the victims of the "wind-bags."

The unsatisfactory condition of psycho-analysis is exemplified in the disputes between followers of the different schools. This is, of course, almost inevitable in the early stages of any doctrine which cannot lay claim to finality. But it should make for more humility in some of its exponents, and prevent them from allowing the uninitiated to infer, that here is a panacea for mental ills. Freud, indeed, here sets them a good example. He evidently realises as everyone does eventually who has laid too much stress on the psychological form of treatment, and has had sufficient experience to disillusion him, that there are still many problems to be solved before we can become optimistic. Nevertheless one cannot help feeling that once again, in this most interesting exposition of his views, his theories outrun the warrant of his facts. But this volume is certainly stimulating.

Freud deals here with the applications of psycho-analysis to the psycho-pathology of everyday life under the heading of "the psychology of errors," to dreams, and to the neuroses. There is a great advantage in this book in that he has not presupposed any knowledge of his theory, and has, therefore, explained his methods in detail—especially in the earlier part. He has, too, limited himself to the topics mentioned, and has not included others, such as sociology and anthropology. In some ways this is an advantage; but, on the other hand, by judicious compression he might have given in the same amount of space a survey of the other fields of investigation. This is certainly a book which anyone anxious to acquire a knowledge of psycho-analysis should possess, especially as in it they have the *ipse dixit* of Freud without the gloss of (perchance) too devoted followers. The translation seems to have been well done.

---

**BIRTH-CONTROL AND POPULATION.** An Essay by Colonel G. T. K. Maurice, C.M.G., C.B.E., A.M.S. (ret.). (London: The Scientific Press, Ltd.; 1922; pp. 56; 2s. 6d.)

It is now more than a century since Malthus called the attention of politicians to the fact that population constantly tends to outgrow the means of subsistence, and is prevented from doing so by "checks," such as prudence, poverty and war. Though this doctrine is indisputable, and was the germ from which Darwin derived "the struggle for existence" of all living things, its fundamental political importance is far from sufficiently appreciated. Col. Maurice holds that for every area there is "a best population." To exceed this is detrimental to well-being, because of the increasing difficulty of food supply, and is one cause of war. If the Germans had limited their birth-rate to the extent of their territory there might have been no great war, and if the Japanese do this, a potential cause of war will be removed. But "man has not yet applied his brains to the breeding of his kind, but has left the matter to chance," and "a time will come when some plan of restraint must be adopted; if not, the human races will be forced into wars of extermination." Emigration, which the author has recently discussed in our columns (The Hospital and Health Review, May, p. 223), is not a satisfactory remedy, as it is the best, rather than the worst, who are lost by the mother country. Neither can the difficulty be surmounted by the importation of food in exchange for manufactured articles. For the new or backward countries are more and more developing manufactures and consuming their food supplies. Thus, if population continues to increase, "a degrading and selfish poverty will press down the community till starvation wipes out the more feeble and gives the remainder space to grow food."

The remedy advocated by the author is birth control, which he defines as "interference with the procreation of a species which makes the number or quality of the births different from what it would be without reasoned intervention." Man controls the birth of his own species, but does it irrationally. The control is in two forms—individual prudential control, which restrains births; public sentimental control (doles, &c.), which tends, but does not purpose, to increase births. As the people above the average practise prudential control, while those below it do not, the effect is to lower the mental and physical average of the community. The practice of birth control is increasing, and the author suggests that it should be directed by the community for the good of the community. "Wisely guided, it would eliminate the unhealthy and feeble, and so raise the average." This essay is a lucid exposition of the problem of population and its bearing on human welfare.

---

**MANUAL OF PHYSIO-THERAPEUTICS.** By Thomas Davey Luke, M.D., F.R.C.S.(Ed.). New and Revised Edition. (Wm. Heinemann, Medical Books, Ltd.; 25s. net.)

The treatment of disease other than by drugs is an aspect of medical practice which does not find, perhaps, so large a place in the medical curriculum as its importance deserves. It is not sufficient for the practitioner to know that such and such a bath is good for this form of disease, or that a sea voyage is useful in that malady. He should have a practical knowledge of the methods employed to obtain the best results, and he should not be ignorant of the effects of climate upon disease. Vaguely to recommend massage, or a stay at some "hydro," without specifying details, is to lay himself open to the charge of being, to say the least, not quite up-to-date in medical science. There is no denying the fact that the public expect their medical advisers to be fully *au courant* with the methods of every cure, legitimate or otherwise, that is served up with their morning paper. In the present volume, which has been largely rewritten, the author deals fully with all the physio-therapeutic measures in common use, including baths, massage, rest, electricity, and diet. A special section is occupied with a description of the Bruce-Sutherland system of medical gymnastics. The English health resorts receive well-merited praise, but the author utters a warning against sending invalids to two varieties of hydropathic establishments—those which are practically indifferent boarding-houses, and those which exist purely for pleasure, and in which there is "no pretence for giving facilities for diet or treatment." In connection with vibratory treatment we are told that "five to ten minutes' vibration over the hepatic area is equal to at least an hour's horse exercise," and, we should imagine, a good deal cheaper too. There is a good account of high-frequency and the Bergonié treatments.

Whether treatment by diet can strictly be called a physio-therapeutic measure is open to question, but the concluding section deals briefly with feeding in health and disease. Some exception may be taken to the statement on p. 420 that the harm done by the abuse of tea as a beverage is "only second to that caused by alcohol." The average British hostess, we would fain believe, does not invariably make it a practice of offering her belated visitor a cup of tea which "would temporarily inhibit the peptic processes of an ostrich."

The numerous illustrations considerably enhance the value of this book.—G. N. M.

---

**THE PRACTITIONER'S MANUAL OF GYNÆCOLOGY.** By A. C. Magian, M.D. 1922. (Wm. Heinemann, Medical Books, Ltd.; 21s. net.) Pp. xiv., 436. With 147 illustrations.

The author has set himself the task of providing a "readable synopsis of some of the principal diseases peculiar to women." He has given the practitioner a practical guide to the diagnosis and treatment of gynæcological affections in general. That he has drawn freely upon the works of well-known authorities does not detract from the worth of the present volume. An anatomical classification is adopted, which possesses many advantages from the point of view of reference. The dangers attendant upon careless introduction of the uterine sound are rightly insisted upon, whilst the once popular pessary comes in for considerable adverse criticism. The use of such supports is being gradually discouraged, except when operative interference of any kind is resented or inadvisable. Failure to diagnose early malignant disease is serious and much to be regretted. The author wisely advocates a "second opinion when dealing with neurotic women complaining of pelvic symptoms." The influence of the endocrine glands upon the sexual organs is adequately discussed, and a good account is given of the principles of operative technique. The operating-theatre, with all its associated equipment, is to be selected in preference to the private house, wherever possible, and the author pertinently remarks that the former place is *not* one in which to smoke! A selection of suitable prescriptions and a chapter on the differential diagnosis of gynæcological affections serve to complete the work.—G. N. M.

---

**MODERN METHODS IN THE DIAGNOSIS AND TREATMENT OF RENAL DISEASE.** By Hugh Maclean, M.D., D.Sc. (Constable; 8s. 6d. net.)

The author of this book is well known for his investigations into the incidence of albuminuria and war nephritis among soldiers, and it was he also who, in conjunction with de Wesselow, introduced the urea concentration test into clinical laboratory practice. In less than one hundred pages he has described the latest methods in use for determining the functional efficiency of the kidneys, as well as for testing the blood in renal disease.

In the absence of any symptoms indicative of kidney trouble the mere discovery of albumin in the urine, unassociated with blood or pus, does not help much in the diagnosis, but the probability is that the condition is not serious. Careful clinical investigation is, however, necessary in all cases to avoid error. The simplicity of the urea concentration test has much to recommend it, and, in the majority of cases, it will be found to give more information than any other method. Of the various dyes used to estimate renal function the most popular one is "phenol red," which is non-toxic and non-irritating.

Of much value is the scheme outlined for the examination of renal patients which is now in use all over the country in the clinics of the Ministry of Pensions. This scheme was evolved by the author, and it has the advantage that as many as thirty to forty patients can be examined by it in one day. The best test for protein in urine is stated to be salicylsulphonic acid, being more sensitive than either the heat or the cold nitric acid test. This able monograph will be welcomed by medical men who desire to become acquainted with the newer methods of estimating renal efficiency.—G. N. M.

---

**RICKETS.** By J. Lawson Dick, M.D. (Ed.), F.R.C.S. (Wm. Heinemann, Medical Books, Ltd.; 25s. net.)

Discussion still rages hotly over the etiology of rickets. Is it a consequence of vitamine deficiency or of defective hygiene? Dr. Lawson Dick is an enthusiastic supporter of the latter view, for which he marshals an imposing array of facts with skill. He admits the fascination of the vitamine theory, but he does not fall under its spell. His material extends back across the centuries and ranges over the whole surface of the globe.—C. E. S.

